# Electro acoustic stimulation of the auditory system: UNICAMP's surgical approach

**DOI:** 10.1590/S1808-86942012000100007

**Published:** 2015-10-20

**Authors:** Guilherme Machado de Carvalho, João Paulo Peral Valente, Alexandre Scalli Mathias Duarte, Éder Barbosa Muranaka, Alexandre Caixeta Guimarães, Marcelo Naoki Soki, Walter Adriano Bianchini, Arthur Menino Castilho, Jorge Rizzato Paschoal

**Affiliations:** a Master's degree in medicine (Otorhinolaryngologist, Otorhinolaryngology and Head & Neck Discipline); b Otorhinolaryngologist (Otologist, Otorhinolaryngology and Head & Neck Discipline); c Otorhinolaryngologist (fellow in Otology, Otorhinolaryngology and Head & Neck Discipline); d Otorhinolaryngologist (fellow in Otology, Otorhinolaryngology and Head & Neck Discipline); e Medical doctor (medical resident in otorhinolaryngology); f Otorhinolaryngologist (Otologist, Otorhinolaryngology and Head & Neck Discipline); g Otorhinolaryngologist (Otologist, head coordinator of the Implantable Prostheses and Cochlear Implant Group, Otology Unit, Otorhinolaryngology and Head & Neck Discipline); h Otorhinolaryngologist (Otologist, head coordinator of the Implantable Prostheses and Cochlear Implant Group, Otology Unit, Otorhinolaryngology and Head & Neck Discipline); i Otorhinolaryngologist (Otorhinolaryngologist, faculty member of the Otorhinolaryngology and Head & Neck Discipline, head of the Otology Unit). Cochlear Implant and Implantable Prostheses Group, Department of Otology. Otorhinolaryngology and Head and Neck Surgery Program – School of Medical Sciences – State University of Campinas - São Paulo, Brazil.

**Keywords:** acoustic stimulation, cochlear implants, hearing disorders, hearing loss, rehabilitation of hearing impaired

## Abstract

A new era has arrived in auditory rehabilitation with the introduction of new technologies such as electroacoustic stimulation (EAS). EAS is indicated for patients with residual hearing at low frequencies and severe or profound hearing loss at high frequencies. These patients have no indication for conventional cochlear implant and have difficulties in adapting to individual sound amplification devices. Preservation of hearing is vital in this process; the surgical technique must be based on this concept.

**Objectives:**

To present the cochlear implant surgical technique with MED-EL Mand FlexEAS to preserve hearing in patients with hearing loss at high frequencies and to maintain low frequency hearing. We are the first institution to carry out this treatment in Brazil.

**Methods:**

A case report of the surgical technique carried out in four patients; the procedure was carried out by the cochlear implant group of a specialized clinical hospital.

**Results:**

The procedures were successful and uneventful.

**Conclusion:**

We described the technique used at our institution for implants using EAS; the surgical technique is complex and includes steps for preservation of hearing.

## INTRODUCTION

Just over a decade ago patients with sensorineural hearing loss had few options for auditory rehabilitation; there were hearing aids or sound amplification devices, electrical stimulation of the cochlear nerve (cochlear implants)^1^, ^2^, ^3^, ^4^, ^5^, and electric stimulation of the brainstem (brainstem implant). Mild or moderate cases may benefit from hearing aids; cochlear implants have been considered the gold standard for rehabilitating severe or profound dysacusis^3^, ^4^, ^6^, ^7^, ^8^. Brainstem implants are an alternative for patients in which cochlear implants are not indicated. Some patients, however, remained in an “intermediate zone” in which hearing aids yielded no benefits and the criteria for cochlear implants were not met^2^, ^3^.

These patients often present sloping hearing loss where low frequency thresholds (up to 1,000 Hz) are preserved and higher frequency thresholds are lower, with severe or profound hearing loss at middle and high frequencies. A new form of therapy arose as the concept of auditory preservation developed and technological advances were made in cochlear implants, namely electric acoustic or hybrid stimulation^2^, ^9^, ^10^, ^11^, ^12^, ^13^.

Knowing about the diseases that cause hearing loss is fundamental to help patients by facilitating and speeding auditory rehabilitation processes^14^. Stimulating the auditory system is essential for better communication^15^.

Christian Von Ilberg developed the concept of electric acoustic stimulation (EAS) in 1999 2, 16, 17, 18. The idea behind EAS is the possibility of synergy by associating conventional cochlear implants (electric stimulation) and individual hearing aids (acoustic stimulation) in the same ear. This opened possibilities for treating patients with residual hearing at low frequencies only, which did not benefit significantly from hearing aids^2^, ^10^, ^12^, ^13^, ^16^, ^17^, ^18^.

The possibility of preserving residual hearing following cochlear implant surgery arose with the work of Lenarz; these patients respond better to hybrid stimulation of hearing – electric (cochlear implant) and acoustic (hearing aid)^3^, ^11^. Benefits occur mainly in discrimination of sound in noisy environments^7^, ^19^, ^20^.

Several surgical techniques for preserving hearing have been described^2^, ^10^, ^18^, ^21^. These techniques are refined by using specific drugs during these procedures, such as topical corticosteroids, antibiotics, and hyaluronic acid^11^. Another important point – already demonstrated in experimental work – is the approach for electrode insertion during surgery^11^, ^22^, ^23^, ^24^. Insertion by a cochleostomy anterior and inferior to the round window, or directly through the round window, are related to less intracochlear trauma compared to the traditional cochleostomy; direct insertion through the round window appears to be the least traumatic approach^1^, ^2^, ^18^, ^21^, ^22^, ^23^.

The intrinsic features of electrodes are important for reducing intracochlear trauma – the shape, length, and flexibility of the electrode arrays also help attain better results^2^, ^10^, ^22^, ^23^, ^24^, ^25^, ^26^. Gantz^27^ recently published a report on eight patients that underwent cochlear implant surgery using a 10 mm electrode (Iowa Nucleus Hybrid Implant); the partial or total preservation rate of hearing was 96%. In 2004, MED-EL (Innsbruck, Austria) launched in the market a new thinner and more flexible 21 mm implant prototype^27^, ^28^. Gstoettner reported high rates of partial or total hearing preservation by using the same prototype^26^, ^27^, ^28^.

The purpose of this study was to present our experience of four patients that underwent cochlear implant surgery with the MED-EL Flex^EAS^ electrode. We describe the surgical technique for EAS with the MED-EL Mand Flex^EAS^ model, carried out by the Implantable Prostheses and Cochlear Implant Group, Otology Unit, of a tertiary care hospital, from March 2010 to May 2011.

## MATERIAL AND METHODS

The protocol for selecting patients was the following:

1 Serial audiometry;
2 Free field audiometry;
3 Otoacoustic emissions (OAE) testing;
4 Speech perception test;
5 Brainstem auditory evoked potential (BAEP);
6 Computed tomography of the temporal bones;
7 Magnetic resonance imaging of the inner ear;
8 Phonoaudiological and psychological evaluation.

**Figure 1:**
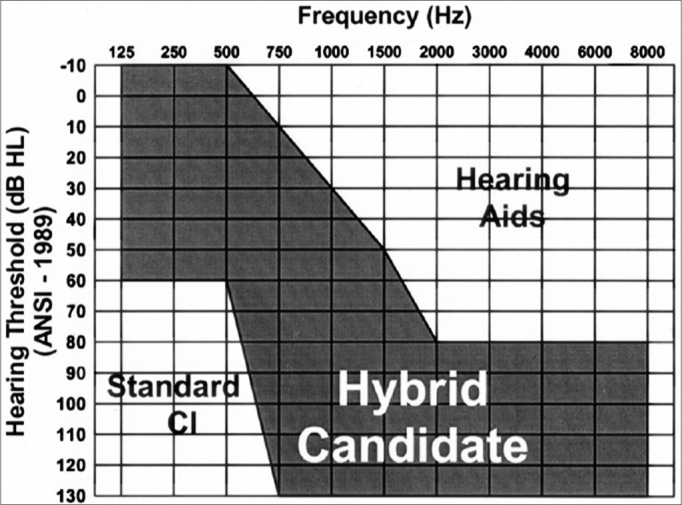
Chart showing an example of an expected audiogram in candidates for hybrid implants.11

The main criteria for indicating the procedure were:

**a)** Stable auditory thresholds within the past two years;
**b)** Speech perception test – (65 dB, with the best possible amplification): < 40%;
**c)** Prior experience with hearing aids;
**d)** Sensorineural type dysacuss^29^ according to the chart below (Figure 1):

### Selection of Patients

Patients were selected based on the abovementioned criteria. All patients were informed about the indication for surgery, possible complications, objectives of therapy, and expected results. Patients were given a free informed consent form after detailed explanations.

### Ethics Committee

The institutional review board approved this study.

### Inclusion criteria

- Age over 18 years.
- Bilateral sensorineural dysacusis with little or no benefit from hearing aids.
- Pure tone thresholds better than 65 dB at 125, 250, and 500 Hz, and worse than 85 dB at frequencies over 1,000 Hz.
- Auditory discrimination for monosyllables below 40% at the best possible sound amplification.
- Stable hearing loss in at least the past two years.

### Assessment

Before surgery, conventional audiometry, speech perception tests with and without hearing aids, OAE (otoacoustic emissions) testing, BAEP (brainstem auditory evoked potential), computed tomography and magnetic resonance imaging of the temporal bones were done in all patients. A psychological evaluation was carried out to discuss expectations about the implants.

Implants were activated 45 days after surgery. Postoperative audiometry and speech perception tests were carried out.

### Implants

**Figure 2:**
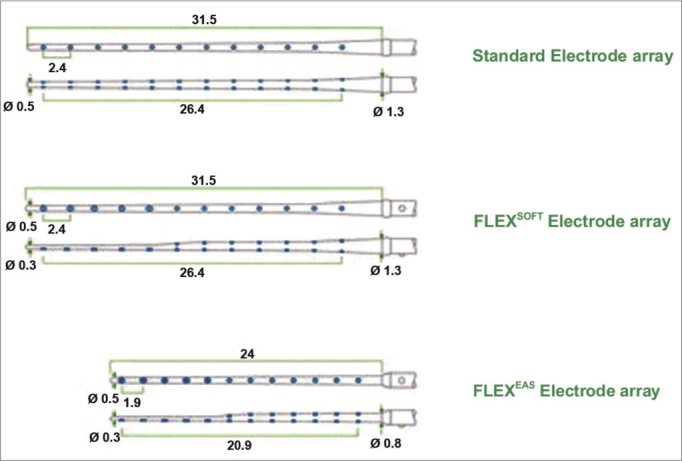
Schematic representation of the Electrode Med-El MAnd FLEXEAS ™(21mm).

**Figure 3:**
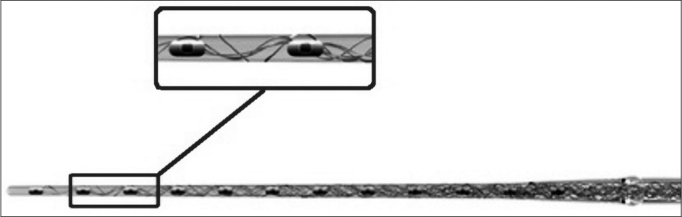
Amplified schematic showing the wave shape of electrodes (sinus) in the silicone sheath with a wire structure.

The implant that was used in these cases was the Med-El MAnd FLEX^EAS^ ^TM^ (21 mm) (Figures 2 and 3)^30^. The purpose of the electrode is to reduce intracochlear trauma during insertion as much as possible; thus, each set of electrodes has a wave-like wire configuration, placed on the lateral wall, and having low channel density (Figure 3).

This project is ideal for insertion through the round window, a technique many otologic surgeons prefer because it is less traumatic. Premolded electrodes are more traumatic and are not easily inserted through the round window; they are therefore inadequate for preserving hearing. Many insertions were done through the scala vestibuli – rather than the scala tympani – with this type of electrode^2^, ^10^, ^22^, ^23^, ^24^, ^25^, ^26^, ^27^, ^28^.

Table 1 summarizes the technical data of the EAS cochlear implant.

**Table 1:** Technical specifications of the Med-El MAnd FLEXEAS cochlear implant

Model: SONATAti100 ELECTRODE FLEX^EAS^	
Material: TITANIUM AND SILICONE	
Removable magnet	NO
Size	Length: 45.7 mm
	Width: 24.8 mm
	Thickness: 5.9 mm
	Weight: 8.6g

Receiver well for the internal unit	Diameter: 24.8 × 17.4 (mm × mm)
	Depth: 2 mm

Electrode array	Perimodiolar: no
	Straight: yes

Dimensions of electrode array	Total length of electrode array: 24 mm
	Length of electrodes that remain within the cochlea: 20.9 mm
	Length of active electrodes: 20.9 mm
	Number of electrodes: 12 channels (19 electrodes – 5 apex channels are not double for residual preservation)
	Apex: electrode number 1 (number of the most apical electrode).
	Base: electrode number 12
	Diameter of electrode array on the apex: 0.3 mm
	Diameter of electrode array on the base: 0.8 mm
	Diameter of the cochleostomy: 1.3 mm
Allow MR	yes
	Technical specifications: 0.2T, 1.0T e 1.5T

Please, fill out the questionnaire and prior instructions from Med El.

### Monitoring the facial nerve (VII cranial nerve)

The 8^th^ cranial nerve is monitored throughout the procedure. Electrodes are attached to the ipsilateral rami of the operated ear on the frontal, zygomatic, buccal, and mandibular areas; there is also a ground electrode attached onto the patient's thorax and a reference electrode (STIM1 +, positive pole) for the stimulatory pen (STIM 1 -, negative pole) that is attached to the sternoclavicular area. We use a NIM-Pulse^TM^ (Nerve Integrity Monitor, Meditronic Xomed^TM^).

### Microscope

Microscopy is essential and has revolutionized otologic procedures. We use a CARL ZEISS GMGH S88 Microscope™ with a camera and a digital video system for recording the procedures.

## RESULTS

### Description of the surgery

A patient is placed on horizontal dorsal decubitus with the head turned to the contralateral side. General anesthesia with orotracheal intubation is done. The operative field is prepared by ample trichotomy, antisepsis with 2% chlorhexidine, and placement of electrodes for monitoring the 8^th^ cranial nerve. The operative field is separated from the rest of the scalp by using micropore^TM^. Antibiotic prophylaxis is done with endovenous cefazolin (50 mg/kg) during induction of anesthesia.

**Figure 4:**
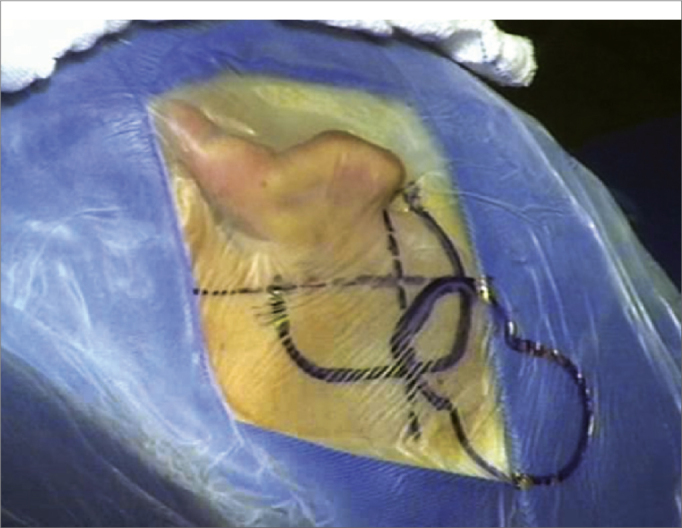
Representative drawing of surgical landmarks to the left (tip of the mastoid, temporal line, retroauricular incision area, area of the internal component, and microphone area with a mock-up).
2 Antisepsis with 0.2% aqueous chlorhexidine, placement of sterile drapes and steri-drape™2 (Figure 1);
3 Rectilinear retroauricular incision and dissection along anatomical planes. Preparation of a “cross” Palva flap (periosteal muscle) raising the four segments of the flap over the subperiosteal plane;
4 Removal of small fragments of fascia and temporal muscle to occlude the cochleostomy;
5 Simple mastoidectomy, identifying the lateral semicircular canal, the short ramus of the anvil, the posterior wall of the outer ear canal, the tegmen timpani, and the lateral sinus. Gathering a small amount of bone dust;
6 Thinning of the posterior wall of the outer ear canal, posterior tympanostomy, preservation of the incus buttress;
7 Preparation of the receiver well for the inner component of the intracochlear EAS on the squamous portion of the temporal bone (well) using a specific mock-up;
8 Irrigation of the cavity with povidone-iodine (10% povidone-iodine / 1% active iodine) for two minutes followed by abundant irrigation with lactated Ringer's solution™ (Figure 5);

1 The main landmarks are marked: tip of the mastoid, temporal line, retroauricular incision line, area of the internal component, and area of the microphone with the help of a mock-up (Figure 4);

**Figure 5:**
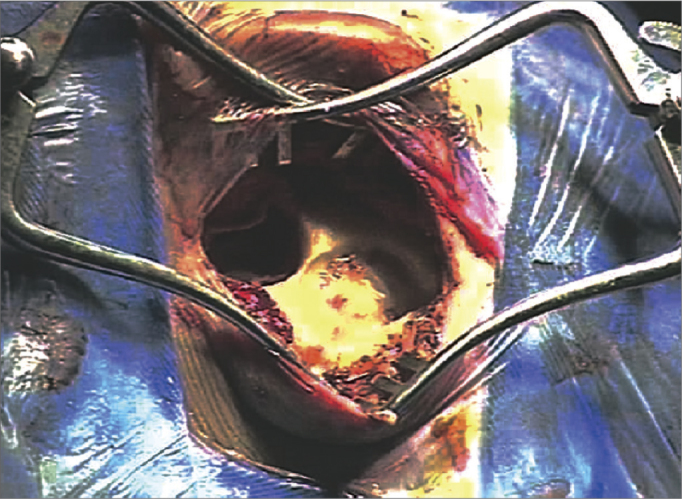
Illustration of preparing the receiver well for the internal component of the EAS cochlear implant in the squamous portion of the temporal bone, with a specific mock-up;
9 Irrigation of the cavity with ciprofloxacin (4mg/ml) for two minutes followed by irrigation with lactated Ringer's solution™ (Figure 6);

**Figure 6:**
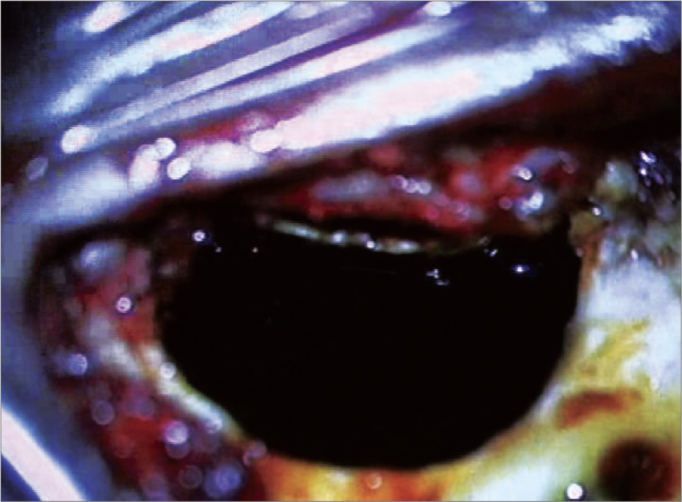
Ilustration of irrigation of the mastoid cavity and middle ear with povidone-iodine solution (10% povidone-iodine / 1% active iodine).
10 Endovenous administration of dexamethasone (8 mg) before approaching the middle ear via a cochleostomy or through the round window;
11 Application of topical triamcinolone (40mg/ml) over the round window;
12 Opening the membrane of the round window; if this approach is impossible, the endosteous is opened by means of a cochleostomy;
13 Positioning the internal component on its well;
14 Preparation of the fascia graft; making a pinhole central orifice to allow the electrode to pass snugly to be placed in the cochleostomy/round window site;
15 Insertion of the electrode slowly and continuously during three minutes through the hole in the graft;
16 Positioning the muscle graft around the electrode to seal the cochleostomy. Placing bone dust to close the posterior tympanotomy;
17 Positioning the ground electrode under the muscle-periosteum flap;
18 Closure with Vicryl^™^ 3.0 sutures on the Palva flap planes and subcutaneous tissue; skin closure with Nylon 4.0;
19 Cleaning of the patient and placing an external compressive dressing;
20 Impedance testing, neural response telemetry (NRT), and a transorbital incidence radiograph are done to confirm the position of the intracochlear electrode.

**Figure 7:**
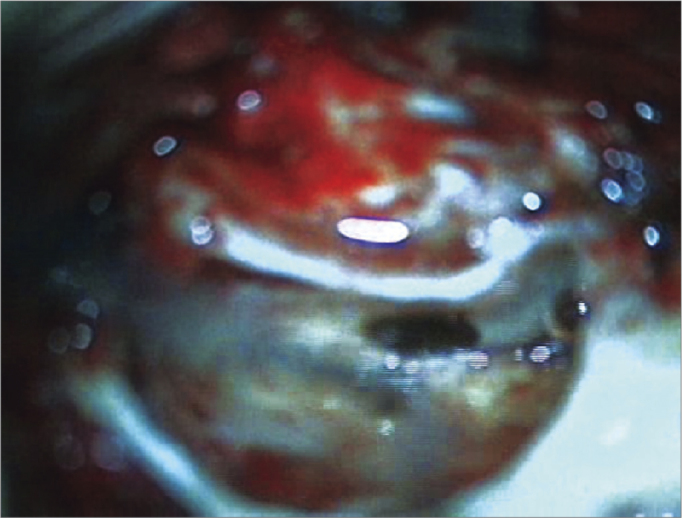
Ilustration of irrigation of the mastoid cavity and the middle ear with antibiotic (4mg/ml ciprofloxacin).

**Note**: it is worth noting that this surgical technique was not created or developed by our tem. We adopted this approach based on the experience that our team had acquired in courses, conferences, and reading the specific medical literature.

## DISCUSSION

Hearing rehabilitation through surgery (cochlear implant) with hearing preservation in patients with hearing remains in the low frequencies becomes a new treatment option^2^, ^9^, ^10^, ^11^.

Electrodes on the lateral wall are less traumatic than premolded electrodes; they are preferable because of this feature and to preserve residual hearing^30^.

Topical medication in the middle ear before placing an implant is a controversial topic; this concern, however, is justified. The purpose of using povidone-iodine and ciprofloxacin is to assure that the operative field remains sterile so that contamination of the inner ear is avoided as much as possible, thereby avoiding possible damage to the inner hair cells. The purpose of corticosteroids topically over the round window and systemically is to reduce intracochlear inflammation, thereby minimizing damage to the round window.

There is a debate in the literature about the topical effect of endovenous drugs in the middle and inner ear^31^. The membranes of the oval and round windows are permeable to several substances; however, there is uncertainty about whether this change in administration route changes or not the pharmacodynamics of these drugs to the point of altering their expected effect in the inner ear^32^, ^33^. The drug formulation, the carrier substance for crossing the membrane, the osmolarity of the solution, the duration of action/exposure of the drug in the inner ear among other factors are important issues for substances that penetrate the membranes to act in the inner ear^31^.

Bird *et al. have shown that the concentration of drugs in the cochlear perilymph* is higher compared to the blood plasma at dosages for endovenous concentrations – such is the case of corticosteroids. These doses were measured in the cochlea and plasma based on equivalent endovenous dosages^34^, ^35^.

The use of lactated Ringer's solution throughout the procedure, especially from the beginning of mastoidectomy, is justified because it is more similar to endolymph, which at least in theory reduces intracochlear trauma.

The ossicular chain should be handled minimally; any disruption among ossicles should be avoided to preserve residual hearing; the ossicular chain can be compromised, which results in loss of conduction and reduced acoustic simulation. Any vibration of the ossicular chain may be conveyed to the inner ear and may cause cochlear injury, sensory loss, and further loss of residual hearing^2^, ^10^, ^18^, ^21^, ^29^.

Several authors consider the approach to electrode insertion – cochleostomy or through the round window – as one of the most important steps to reduce intracochlear damage^29^. Burring and exposure of the inner ear should be done delicately. Cochleostomy increases the risk of damage to the inner ear because burring in the cochleostomy site may cause fluid-mediated injury due to vibration of the periosteum. There may also be damage to soft tissues (spiral ligament, vascular stria, basilar membrane, Corti organ, Reissner membrane, etc.) and to bone (spiral bone lamina, modiolus, Rosenthal's canal, etc.). The vascular stria is commonly damaged in conventional cochleostomy, which explains its worse results in hearing preservation. Opening the periosteum in cochleostomy and through the round window allows perilymph to exit, which disrupts the intracochlear hemostasis (especially the electrochemical gradient); there is also the possibility of trauma by inadvertent suction^29^.

Therefore, insertion of the electrode through a cochleostomy anterior and inferior to or directly through the round window is currently the method of choice; this approach increases the certainty of accessing the scala tympani and has a lower potential for cochlear trauma^29^.

Electrodes should be inserted slowly, continuously, and at a constant rate. Rapid electrode insertion may cause fluid-mediated injury. Care should be taken not to introduce contaminants (blood, bone dust, secretions) into the intracochlear compartment, which would increase local inflammation. Electrodes should fit snugly, but without resistance; care should be taken not to suction endolymph. There is an exponential increase in resistance to progression after the electrode is inserted by about 15 mm. The electrode specifications, its material (it should be as inert as possible to minimize foreign body inflammation), flexibility, characteristics of the internal arrays, diameter, length, and others, are also important factors^29^.

The cochleostomy is sealed with a temporal fascia graft, which should not be too large so as not to affect the mobility of the ossicular chain and to avoid inflammation from altering the middle ear homeostasis^29^.

The steps of surgery were planned to preserve residual hearing and to reduce cochlear injury as much as possible.

Although much has been said about EAS, this mode of therapy is rarely undertaken in Brazil. Our unit has pioneered this procedure in this setting, and not many such procedures have been done. We point out that cochlear manipulation surgery aiming to preserve hearing is difficult.

## CONCLUSION

We described the technique for EAS implants as applied to the cases operated at our unit. This technique differs from conventional cochlear implant routine by including steps aiming at hearing preservation. The technique is complex and has nuances that make it difficult to carry out adequately.

We believe that cochlear implants with hearing preservation opens a new era in otology and the rehabilitation of patients with hearing loss.
